# Genome-Wide Transcriptome Analysis Revealing the Genes Related to Sugar Metabolism in Kernels of Sweet Corn

**DOI:** 10.3390/metabo12121254

**Published:** 2022-12-12

**Authors:** Bin Chen, Shouli Feng, Junfeng Hou, Ying Zhu, Fei Bao, Hailiang Han, Heping Tan, Guiyue Wang, Fucheng Zhao

**Affiliations:** 1 Institute of Maize and Featured Dryland Crops, Zhejiang Academy of Agriculture Sciences, Dongyang 322100, China; 2 College of Agriculture & Biotechnology, Zhejiang University, Hangzhou 310030, China; 3 Institute of Virology and Biotechnology, Zhejiang Academy of Agricultural Sciences, Hangzhou 310022, China

**Keywords:** sweet corn (*Zea mays* var. *saccharate* Sturt), kernel sugar metabolism, genome-wide transcriptome analysis

## Abstract

Sugar metabolism influences the quality of sweet corn (*Zea mays* var. *saccharate* Sturt) kernels, which is a major goal for maize breeding. In this study, the genome-wide transcriptomes from two supersweet corn cultivars (cv. Xuetian 7401 and Zhetian 11) with a nearly two-fold difference in kernel sugar content were carried out to explore the genes related to kernel sugar metabolism. In total, 45,748 differentially expressed genes (DEGs) in kernels and 596 DEGs in leaves were identified. PsbS, photosynthetic system II subunit S, showed two isoforms with different expression levels in leaf tissue between two cultivars, indicating that this gene might influence sugar accumulation in the kernel. On the other hand, hexokinases and beta-glucosidase genes involved in glycolysis, starch and sucrose metabolism were found in developing kernels with a genome-wide transcriptome analysis of developing kernels, which might contribute to the overaccumulation of water-soluble polysaccharides and an increase in the sweetness in the kernels of Xuetian 7401. These results indicated that kernel sugar accumulation in sweet corn might be influenced by both photosynthesis efficiency and the sugar metabolism rate. Our study supplied a new insight for breeding new cultivars with high sugar content and laid the foundation for exploring the regulatory mechanisms of kernel sugar content in corn.

## 1. Introduction

Maize is an important food, energy and forage crop in the world [1,2]. Statistically, the world trade volume of corn exceeded 182 million tons for more than USD 39.576 billion in 2019 [3]. Sweet corn (*Zea mays* var. *saccharate* Sturt) is also a popular vegetable in Europe and America due to its elite characteristics, such as rich nutrition, kernel sugar content, etc. [4]. According to statistics from the United States Department of Agriculture, the value of sweet corn in the United States is approximately USD 743 million, most of which is used for fresh food and the remainder for canning or frozen processing [5].

Sweet corn evolved from mutants that had gene defects in starch biosynthesis and resulted in an altered carbohydrate composition, increasing sugar and decreasing starch in the endosperm. Several genes in the starch biosynthetic pathway have been proven to increase kernel sugar content in the endosperm of sweet corn [6,7,8,9,10]. Sweet corn is divided into normal sweet corn, super sweet corn and sugary enhancer corn depending on their mutated genes [11]. In normal sweet corn, the sugar1 (*sul*) and *su2* genes are mutated, and the kernel sugar content is 1-3 times greater than that of normal maize at the milking stage (R3), as the mutant of sugar1 (*su1*), a starch-debranching enzyme, has lost its function. Regulated by recessive genes such as shrunken (*sh1*, *sh2* and *sh4*), which encodes the large regulatory subunit of heterotetrametric ADP-glucose pyrophosphorylase (AGPase), the amount of sucrose in the kernels of supersweet corn at the R3 stage is greater than water-soluble polysaccharides (WSPs). When *su1se* is double recessive, sugary enhancer corn exhibits the characteristics of both normal and supersweet corn, as sugary enhancer (*se*) is a recessive modifier of *su1* gene expression, resulting in the sucrose and WSP contents in the kernel both being high at the milking stage [12].

There are different endosperm recessive mutations in sweet corn—such as *su1*, *su2*, *sh1*, *sh2*, *sh4*, *bt1* and *bt2*—that reduce starch synthesis activities, increase sucrose accumulation and produce the enhancement phenotype of sweet components in kernels [13,14]. However, shrunken2 (*sh2*) and sugary enhancer1 (*se1*) are endosperm mutations of increasing importance in the sweet corn industry due to their superior eating quality [15]. Supersweet corn carries an *sh2* mutant that maintains a high sugar level with a stable starch accumulation rate. *sh2* encodes the large subunit of the heterotetrametric ADP-glucose pyrophosphorylase (AGPase) present in the maize kernel endosperm, which is the rate-limiting enzyme in the starch synthesis pathway [16,17]. The activity of AGPase is inhibited by the mutation of *sh2* and results in the accumulation of sugar in the kernel [13]. Sugary enhancer corn exhibits the phenotypes of both normal sweet corn and super sweet corn as the *su* gene is co-expressed with *su1* [12].

Xuetian 7401 and Zhetian 11 are new supersweet corn cultivars that are both widely planted in Zhejiang Province, China, and the kernel of Xuetian 7401 is much sweeter than that of Zhetian 11. Despite both cultivars carrying an *sh2* mutant, the mechanism of the kernel sugar metabolism of the cultivars is not clear. In order to explore the mechanism, time-resolved transcriptomes of the kernels of Xuetian 7401 and Zhetian 11 on different days after pollination (DAP) were sequenced and analyzed. The transcriptomes of the leaves of the two cultivars at the R3 stage were also analyzed. 

## 2. Materials and Methods

### 2.1. Field Planting and Sampling of Sweet Corn

Xuetian 7401 and Zhetian 11 were planted at Tangxi village, Chengdong Street, Dongyang County, Zhejiang Province, China (120.32° E, 29.27° N). Zhetian 11 was planted before Xuetian 7401 because of its longer growth period, and both cultivars were pollinated on June 10. The main ears of each cultivar were sampled at 10, 14, 18, 22, 26 and 30 DAP. The first leaves under the main ears of Xuetian 7401 and Zhetian 11 were sampled at the R3 stage. 

### 2.2. Determination of WSP in Developing Kernels

The content of WSP in maize kernels was determined in triplicate according to the methods described by Muir et al. [18] and modified properly in this study. Sugar standards included D-fructose, D-glucose, sucrose, D-sorbitol, D-mannitol, D-lactose, kestose, nystose, D-raffinose, D-maltose and stachyose (>99% pure, Shanghai ZZBIO Co., LTD, Shanghai, China). All sugar standards were kept dry in a desiccator containing silica gel. Standard solutions were prepared via water purified by a Millipore Milli-Q water purification system (Millipore, Milford, MA). Acetonitrile and methanol (Moke, chromatographically pure) were used. Ultra-performance liquid chromatography was carried out using an ACQUTY UPLC consisting of an ACQUITY ELSD detector and an Empower workstation (Waters, USA). In total, 1.0 g of frozen–dry fresh kernel was dissolved in 80 mL of ddH_2_O and heated at 80 °C with shaking for 15 min. The solution was kept at a constant volume pf 100 mL with ddH_2_O after cooling. In total, 10 mL was centrifuged at 6500× *g* for 5 min, and then, 2 mL of supernatant was filtered by an OASIS HLB (0.22 μm). An Insertsil NH2 column was used for the chromatographic procedure. The mobile phase consisted of pump acetonitrile, pump water and gradient elution (acetonitrile: water = 90:10, 80:20, 70:30). The pump flow rate was 1 min/mL; the column temperature was 35 °C; and the injection column was 10 μL. The evaporation temperature of the chromatographic eluent was 55 °C, and the sprayer was 36 °C.

### 2.3. RNA Extraction and RNA-Seq

The total RNA of the samples was extracted by a Universal Plant Total RNA Extraction Kit (BioTech, Beijing, China). In total, 1 μg of total RNA was prepared for cDNA libraries using a cDNA-PCR Sequencing Kit (SQK-PCS109, Oxford Nanopore Technologies, Oxford, UK) protocol provided by Oxford Nanopore Technologies (ONT). Briefly, the template switching activity of reverse transcriptase was enriched for full-length cDNAs, and we added defined PCR adapters directly to both ends of the first-strand cDNA. In addition, following cDNA PCR for 14 circles with a LongAmp Tag (NEB), the PCR products were then subjected to ONT adaptor ligation using T4 DNA ligase (NEB). Agencourt XP beads were used for DNA purification according to the ONT protocol. The final cDNA libraries were added to FLO-MIN109 flowcells and run on the PromethION platform at the Biomarker Technology Company (Beijing, China).

### 2.4. DEGs Identification

After read processing, removing redundancies, finding fusion transcripts, structure analysis, transcription factor prediction, lncRNA analysis and gene functional annotation, differential expression analysis of two conditions/groups was performed using the DESeq2 R package (1.6.3) [19]. DESeq2 provides statistical routines to determine differential expression in digital gene expression data using a model based on negative binomial distribution. The resulting *P* values were adjusted using Benjamini and Hochberg’s approach to control the false discovery rate. Genes with an FDR < 0.01 and foldchange ≥ 2 found by DESeq2 were assigned as being differentially expressed. Functional enrichment analysis mainly included GO enrichment analysis [20] and KEGG pathway enrichment analysis [21,22]. Finally, protein–protein interactions were analyzed and visualized [23]. 

### 2.5. Time Course Analysis

maSigPro was employed for time course analysis based on gene expression. To compare the differential genes between Xuetian 7401 and Zhetian 11, a multiple-series time course experiment was performed. The significant genes were selected using the function p.vector() with FDR < 0.05. The significant differences between Xuetian 7401 and Zhetian 11 were found using the T.fit() function with default parameters. Finally, the cluster genes were obtained using the get.siggnens() function with rsq (R-squared of the regression model) > 0.05. 

### 2.6. Weighted Correlation Network Analysis

Weighted correlation network analysis was performed using the WGCNA R package. The genes with the top 75% of median absolute deviation (MAD) and MAD greater than 0.01 were selected for WGCNA analysis. The D-fructose, D-glucose, sucrose and total sugar contents in the samples were used as the trait data. After using pickSoftThreashold, power 6 was used to construct the unsigned co-expression network.

### 2.7. RT-qPCR

Primers of seven randomly selected DEGs were designed using the Primer Premier 5 Design Program (Premier Biosoft International, Palo Alto, CA, USA) to verify the quality of RNA-seq data (Table 1). The RNA was extracted using a general plant total RNA extraction kit (RP3301, BioTech, Beijing, China), and the first-strand cDNA synthesis of all samples was carried out with a HiScript III 1st Strand cDNA Synthesis Kit (+gDNA wiper) (R312-01, Vazyme, Nanjing, China). The concentration of cDNA was adjusted to 100 ng/μL. qRT-PCR was carried out using the ABI QuantStudio 7 (Thermo Fisher, Waltham, MA, USA). The reaction mixture (5 μL) contained 2.5 μL of 2 × ChamQ Universal SYBR qPCR Master Mix, 0.1 μL of forward primer, 0.1 μL of reverse primers, 1 μL of cDNA and 1.3 μL of ddH_2_O. The thermocycle parameters were as follows: initial polymerase activation for 30 s (s) at 95 °C and then 40 cycles of 30 at 95 °C; 60 s at 58 °C; and 30 s at 72 °C. Relative expression levels were calibrated and normalized to the level of the *Cyanate* gene. 

### 2.8. Statistical Analysis

Analysis of variance of the experimental data was performed using SPSS Statistics 25 (IBM, Armonk, NY, USA). Significant treatment effects were determined based on the magnitude of the F value (*p* = 0.05). All tests were conducted with triple repeats.

## 3. Results

### 3.1. Dynamic Changes of WSP Contents in Developing Kernels

The dynamic changes of WSP content in the developing kernels of Xuetian 7401 and Zhetian 11 were determined with UPLC-ELSD in this study. Only D-fructose, D-glucose, D-sorbitol and sucrose were determined in all samples, and the content of D-sorbitol was much less than that of the other three sugars. Thus, we mainly analyzed the dynamic changes in D-fructose, D-glucose and sucrose in the developing kernels, and the total kernel sugar content of the kernels was the sum of three sugars. 

**Figure 1:**
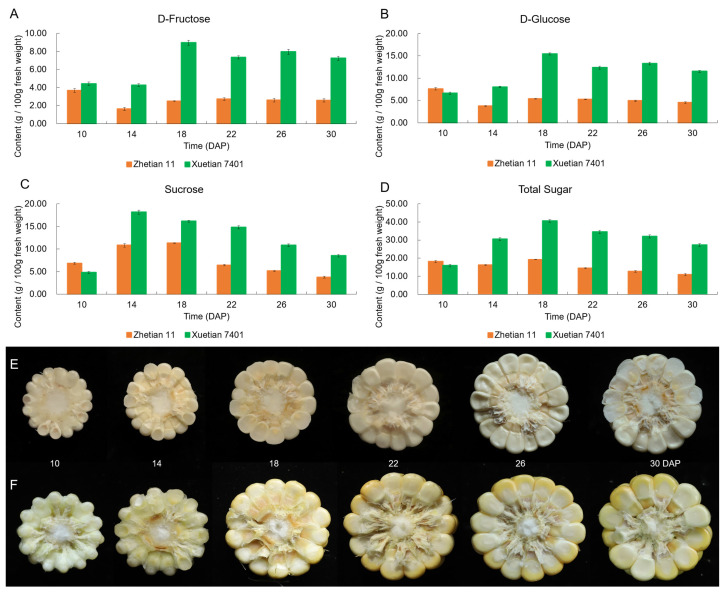
Dynamic changes in the three sugar contents in developing kernels of Xuetian 7401 and Zhetian 11. (**A**–**C**) Dynamic changes in D-fructose (**A**); D-glucose (**B**); sucrose (**C**); and total sugar (**D**) in developing kernels of Xuetian 7401 and Zhetian 11. (**E**,**F**) Cross-sections of the developing ears of Xuetian 7401 (**E**) and Zhetian 11 (**F**). Data in (**A**–**C**) are mean ± SE.

Generally, the contents of the three sugars in Xuetian 7401 were always higher than those in Zhetian 11, except at 10 DAP (Figure 1). The contents of D-fructose and D-glucose in the kernels of Xuetian 7401 after 14 DAP were more than twice that of Zhetian 11. The sucrose content of Xuetian 7401 reached a peak at 14 DAP, while D-fructose and D-glucose showed peaks at 18 DAP. However, Zhetian 11 did not exhibit obvious peaks at 10 to 30 DAP. Interestingly, the D-fructose and D-glucose contents rose again at 26 DAP in Xuetian 7401, while Zhetian 11 basically maintained a slow decline after 18 DAP. 

### 3.2. Quality Analysis of RNA-Seq Data

The RNA-Seq data were qualified for analysis. The original data format of the Nanopore sequencing output data was the second-generation fast5 format, which contained all original sequencing signals. After base calling performed by the Guppy software in the MinKNOW2.2 package, data in the fast5 format was converted to the fastq format for subsequent quality control analysis. After filtering short sequences and the low-quality reads of raw fastq data, the total clean data was obtained. For 36 kernel samples, at least 1.8 million reads were obtained for each sample with an N50 of no less than 995 bp and a mean length of no less than 1026 bp (Table S1), of which more than 98.36% of the sequences were homological to the reference genome. For leaf samples, at least 1.5 million reads were obtained for each sample, with an N50 of no less than 1351 bp and a mean length of no less than 1221 bp (Table S1), of which more than 97.16% of the sequences were homological to the reference genome.

**Figure 2:**
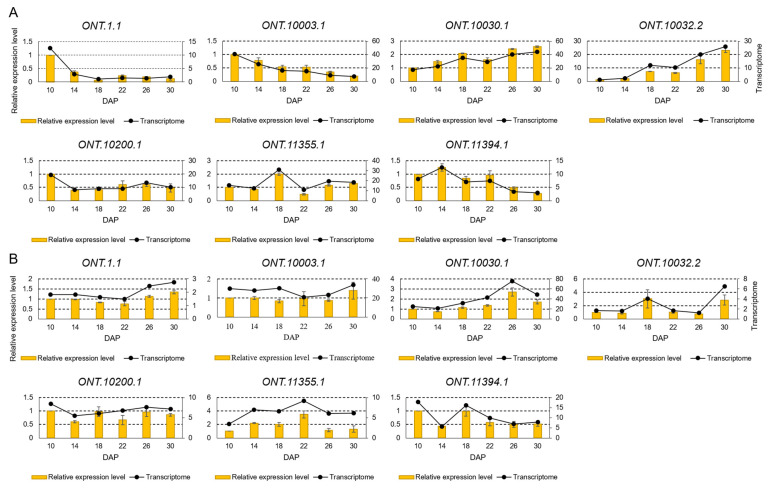
Validation of expression levels of seven randomly selected DEGs in developing kernels of Xuetian 7401 and Zhetian 11. Gene expression levels determined by qRT-PCR are represented by a column and RNA-Seqs are represented by a line. (**A**) Xuetian 7401; (**B**) Zhetian 11. The data of the qRT-PCR are mean ± SE.

The relative expression levels of the seven DEGs to *Cyanate* gene in the developing kernels of Xuetian 7401 and Zhetian 11 were determined to verify the quality of the RNA-seq data. The relative expression levels of the seven DEGs in the kernels of both cultivars were all consistent with the transcriptome data (Figure 2). Principal components analysis (PCA) showed that all samples exhibited good repeatability (Figure S1). The results suggested that the RNA-Seq data in this study were highly qualified. 

### 3.3. RNA-Seq Analysis and DEG Statistics

In order to determine the DEGs related to sugar accumulation in kernels, the transcriptome analysis of kernels contained two dimensions (16 sets): 10 DAP vs. other dates of the same cultivar and Xuetian 7401 vs. Zhetian 11 on the same date. In total, 45,748 DEGs were identified in all analyses of the kernels, with 23,928 upregulated genes and 21 820 downregulated genes (Table 2). It is not difficult to see that the DEG numbers in the developing kernels of Xuetian 7401 were greater than those of Zhetian 11. Meanwhile, the DEG number of Zhetian 11 vs. Xuetian 7401 at 18 DAP was much greater than any other date. On the contrary, the DEG numbers of Zhetian 11 (10 DAP) vs. Zhetian 11 (14 DAP) and Zhetian 11 (22 DAP) vs. Xuetian 7401 (22 DAP) were the smallest in each dimension. In total, 596 DEGs with 310 upregulated genes and 286 downregulated genes were found in the transcriptome analysis of the leaves of Zhetian 11 vs. Xuetian 7401.

### 3.4. RNA-Seq Analysis and DEG Statistics

**Figure 3:**
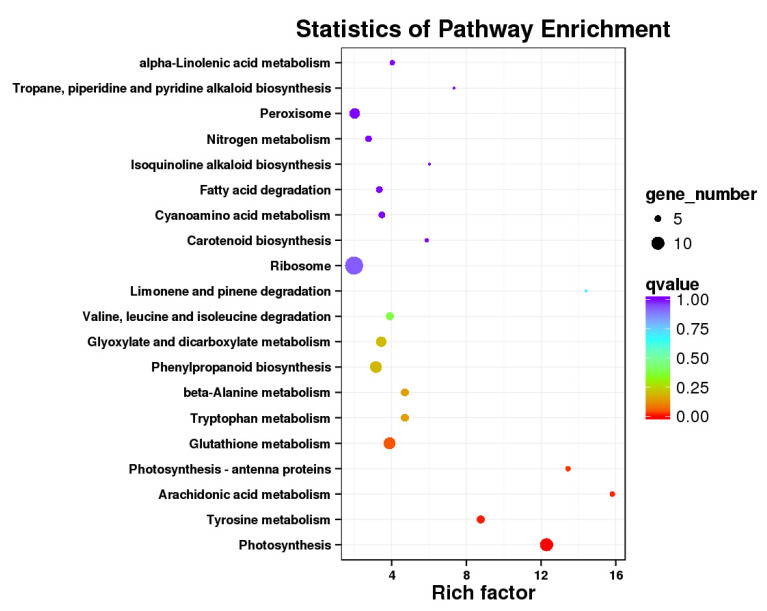
Scatterplot of KEGG pathway enrichment of DETs in leaves. Each circle in the figure represents a KEGG pathway; the Y-axis presents the name of the pathway; and the X-axis presents the enrichment factor, indicating the ratio of the proportion of transcripts in the DETs that are annotated to the pathway to the proportion of transcripts in all transcripts that are annotated to the pathway. The higher the enrichment factor, the more significant the enrichment level of the DETs is in this pathway. The color of the circle represents the q-value; the q-value is the *p*-value after multiple hypothesis tests and corrections. The smaller the q-value is, the more reliable the enrichment significance of the DETs in this pathway. The size of the circle indicates the number of transcripts enriched in the pathway; the larger the circle, the more transcripts.

In order to explore the biological metabolism and metabolic network that might be related to sugar accumulation in kernels, GO and KEGG enrichment analyses of the DEGs were conducted. According to the GO enrichment of the leaf DEGs (Figure S2), the secondary functions of cell killing and protein tag exhibited significant differences. However, the function of cell killing and protein tag seemed to not be involved in sugar accumulation. Similarly, no pathway that might influence sugar accumulation in kernels was found in the KEGG enrichment result (Figure S3). However, the photosynthesis pathway was found in the KEGG enrichment of the leaves’ different expression transcripts (DETs), with a low q-value suggesting a divergence in photosynthesis in the two cultivars, which might influence the accumulation of sucrose in the plants (Figure 3). Interestingly, two isoforms of *ZEAMMB73_Zm00001d042697*, annotated as photosynthetic system ii subunit S (PsbS), were found in the leaves of Xuetian 7401 and Zhetian 11. Although two isoforms could be found in both cultivars, the expression levels were diverse. The expression level of *isoform_1* in Xuetian 7401 was more than 200 times greater than that of Zhetian 11. Meanwhile, the expression level of *isoform_2* was only one-fifth of Zhetian 11. The alignment of the sequences of the two transcripts showed that the sequence of *isoform_1* lacked two regions (20 bp for each region) compared with *isoform_2*, and the other regions were 100% homological. The divergences of the two transcripts of *PsbS* might be involved in sugar accumulation in kernels.

**Figure 4:**
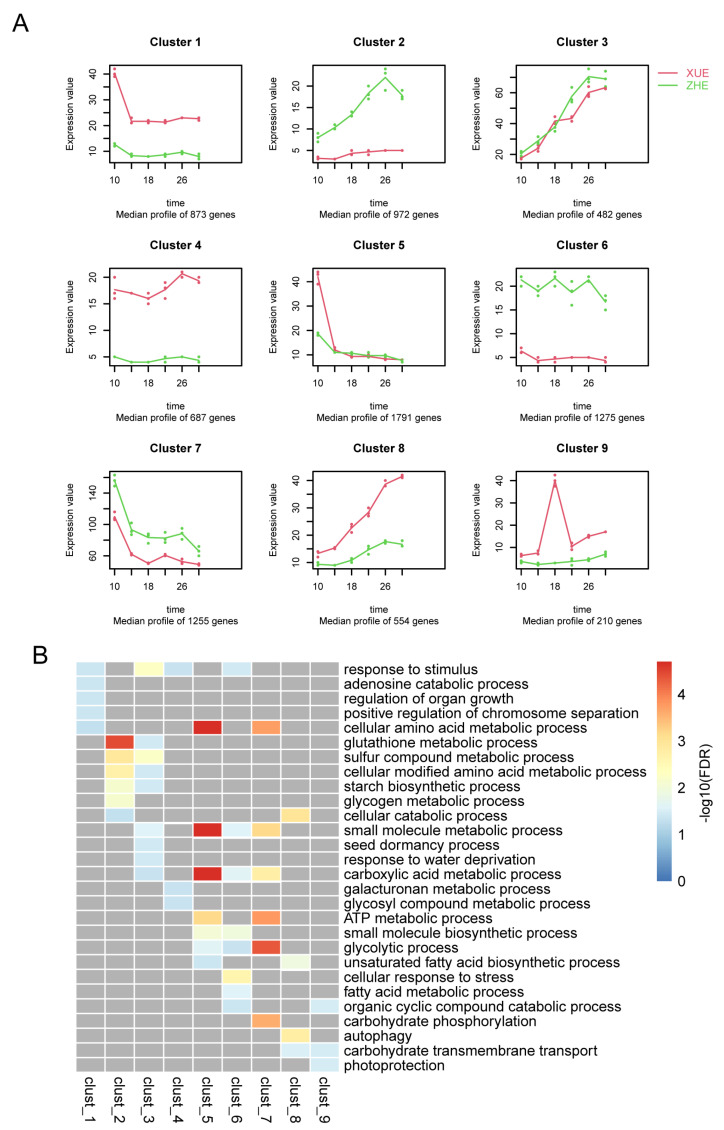
Time-resolved analysis of the developing kernels of Xuetian 7401 and Zhetian 11. (**A**) The DEGs were clustered according to the dynamic expression modes and (**B**) enriched in the KEGG pathways. The red lines in (**A**) reflect the expression levels of Xuetian 7401 genes, and the green ones reflect Zhetian 11 genes.

As it was too complex to analyze the dynamic changes in a single gene of the kernel transcriptomes, the total genes were calculated as differentially expressed and clustered according to the time course analysis, and then, nine clusters were identified (Figure 4A). In clusters 1, 4, 8 and 9, the expression levels of the genes of Xuetian 7401 were always higher than those of Zhetian 11 from 10 DPA to 30 DAP. However, the genes in clusters 2, 6 and 7 were the reverse. In other clusters, the expression levels of both cultivars were interlaced. The genes in each cluster were enriched in KEGG (Figure 4B). 

The expression value of the genes of Xuetian 7401 in cluster 1 at 10 DAP were nearly twice those on other dates. Genes in cluster 1 were enriched in the KEGG pathways in response to stimulus, the adenosine catabolic process, the regulation of organ growth, the positive regulation of chromosome separation and the cellular amino acid metabolic process.

The expression value of the genes of Zhetian 11 reached a peak at 26 DAP and then decreased at 30 DAP. Genes in cluster 2 were enriched in the KEGG pathways in response to the glutathione metabolic process, the sulfur compound metabolic process, the cellular modified amino acid metabolic process, the starch biosynthetic process, the glycogen metabolic process and the cellular catabolic process. The starch biosynthetic process and glycogen metabolic process pathways might be involved in sugar accumulation in kernels.

The expression values of cluster 4 genes in Xuetian 7401 were approximately three times greater than those of Zhetian 11. Genes in cluster 4 were enriched in the KEGG pathways in response to stimulus, the galacturonan metabolic process and the glycosyl compound metabolic process.

The expression values of cluster 6 genes of Zhetian 11 were approximately three times greater than those of Xuetian 7401. Genes in cluster 6 were enriched in the KEGG pathways in response to stimulus, the small molecule metabolic process, the carboxylic acid metabolic process, the small molecule biosynthetic process, the glycolytic process, cellular response to stress, the fatty acid metabolic process and the organic cyclic compound catabolic process. The glycolytic process pathway might influence sugar accumulation in kernels.

In general, the expression values of cluster 7 genes in both cultivars decreased from 10 DAP to 30 DAP; meanwhile, their expression levels in Zhetian 11 were always higher than those of Xuetian 7401 at all dates. Genes in cluster 7 were enriched in the KEGG pathways in response to the cellular amino acid metabolic process, the small molecule metabolic process, the carboxylic acid metabolic process, the ATP metabolic process, the glycolytic process and carbohydrate phosphorylation. The ATP metabolic process and the glycolytic process might be involved in sugar accumulation in kernels.

In contrast with cluster 7, the expression values of both cultivars in cluster 8 generally increased from 10 DAP to 30 DAP; meanwhile, the expression levels of Xuetian 7401 were always higher than those of Zhetian 11. Genes in cluster 8 were enriched in the KEGG pathways in response to the cellular catabolic process, the unsaturated fatty acid biosynthetic process, autophagy and carbohydrate transmembrane transport.

The number of genes in cluster 9 was the smallest in all clusters. The expression values of the genes of Xuetian 7401 at 18 DAP were much higher than those on other dates; meanwhile, the expression levels of the genes of Xuetian 7401 were always higher than those of Zhetian 11. Genes in cluster 9 were enriched in the KEGG pathways in response to the organic cyclic compound catabolic process, carbohydrate transmembrane transport and photoprotection.

In terms of KEGG pathways, genes involving the pathways of the adenosine catabolic process, the regulation of organ growth, the regulation of organ growth, the positive regulation of chromosome separation, the positive regulation of chromosome separation, the galacturonan metabolic process, the glycosyl compound metabolic process, carbohydrate phosphorylation, autophagy, carbohydrate transmembrane transport and photoprotection in Xuetian 7401 were more upregulated than those in Zhetian 11. Inversely, genes involving the pathways of the glycogen metabolic process, the cellular response to stress and the fatty acid metabolic process in Xuetian 7401 were more downregulated than those in Zhetian 11. In addition, genes involving the glycolytic process pathway—which directly plays an important role in sugar accumulation and metabolism in the kernels of Xuetian 7401—were observed to be significantly downregulated in clusters 6 and 7. However, the pathway was also enriched in cluster 5, although the -log_10_(FDR) of cluster 5 was lower than those of clusters 6 and 7. The expression values of the genes of both cultivars in cluster 5 were interlaced, except at 10 DAP. The genes of Xuetian 7401 in cluster 5 at 10 DAP were more upregulated than those of Zhetian 11. The KEGG enrichment results revealed which pathways took part in sugar accumulation in the developing kernels.

**Figure 5:**
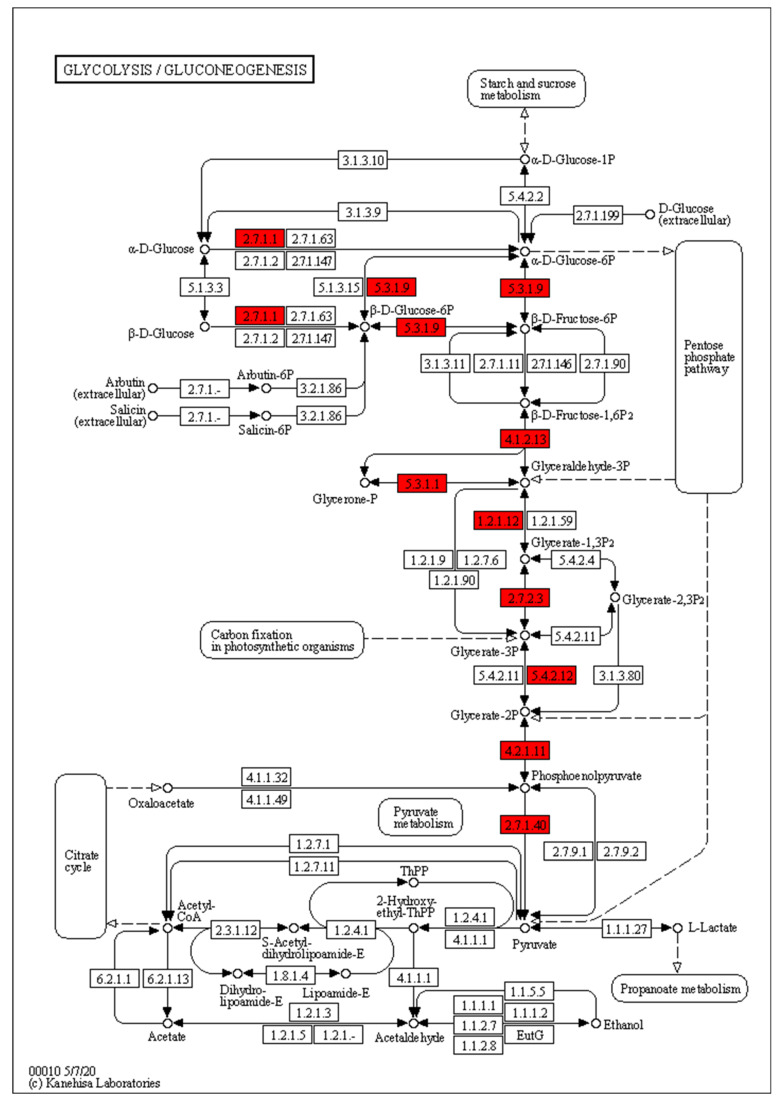
Genes of cluster 6 and cluster 7 involved in the glycolytic process pathway. The genes of cluster 6 and cluster 7 in the pathway are marked in red.

In order to explore the genes involved in sugar accumulation in the kernels, we analyzed the seventeen genes of clusters 6 and 7, which were enriched in the glycolytic process pathway. The genes were mainly divided into nine functions with annotation, including enolase, phosphoglycerate kinase, glyceraldehyde 3-phosphate dehydrogenase, fructose-bisphosphate aldolase (class I), 2,3-bisphosphoglycerate-independent phosphoglycerate mutase, hexokinase, pyruvate kinase, triosephosphate isomerase (TIM) and glucose-6-phosphate isomerase. These genes tended to participate in several reactions in the glycolytic process pathway, such as hexokinase and glucose-6-phosphate isomerase (Figure 5). Since the substrates of the glycolysis process pathway are monosaccharides, such as D-glucose and D-fructose, the upregulation of these genes in the glycolysis process pathway in the kernels of Zhetian 11 implied accelerated substrate consumption. In addition, hexokinases encoded by *Zm00001e019856* also play roles in other pathways involving the metabolism of sugars, such as fructose, and mannose metabolism, such as starch and sucrose metabolism, suggesting that the upregulation of these genes might not only influence the glycolysis process. In summary, the expression levels of the genes in cluster 6 and cluster 7 involving the glycolysis process were more upregulated in the kernels of Zhetian 11 than those in Xuetian 7401, suggesting that the consumption rate of D-fructose, D-glucose and other sugars in Zhetian 11 might be higher and resulting in the divergence of the sugar content in the kernels of these two cultivars.

### 3.5. Identification of Genes Related to Sugar Accumulation via Weighted Correlation Network Analysis (WGCNA)

In order to identify the correlated DEGs that regulated sugar accumulation in the kernels during development, we used WGCNA to analyze gene expressions in all 36 samples collected from 10 DAP to 30 DAP. After removing the genes with low fluctuation expressions, the genes were subjected to a pairwise correlation analysis regarding gene expression and sorted into different modules. 

**Figure 6:**
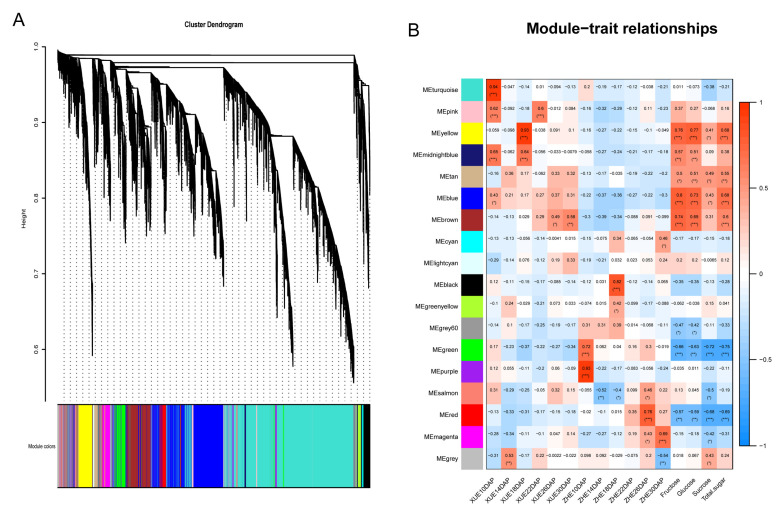
Network analysis of a dendrogram showing modules identified using WGCNA. (**A**) Hierarchical cluster tree showing co-expressing modules. (**B**) Heatmap of module–sugar weight correlations and corresponding *p*-values. * in (**B**) denotes *p* < 0.01, ** denotes *p* < 0.001 and *** denotes *p* < 0.0001.

Eighteen co-expression networks were constructed (Figure 6A,B), and MEyellow, MEmidnightblue, MEtan, MEblue and MEbrown modules exhibited a significant positive correlation with the D-fructose and D-glucose contents (Figure 6B). Interestingly, these modules were more relevant for Xuetian 7401. For example, the connectivity between MEyellow and XUE18DAP reached 0.93, with *p* < 0.0001; meanwhile, MEyellow did not exhibit any significant correlations with Zhetian 11. As the D-fructose and D-glucose contents in the kernels of Xuetian 7401 were all significantly higher than those in Zhetian 11, MEyellow, MEmidnightblue and MEbrown modules were selected and analyzed as the key modules positively associated with kernel sugar content. In addition, MEgreen and MEred modules exhibited negative correlations with all sugars (Figure 6B) and a positive correlation with Zhetian 11. As the connectivity between MEred and ZHET26DAP reached 0.76, with *p* < 0.0001, it was taken to be the module negatively related to kernel sugar content, and functional analysis was carried out.

**Figure 7:**
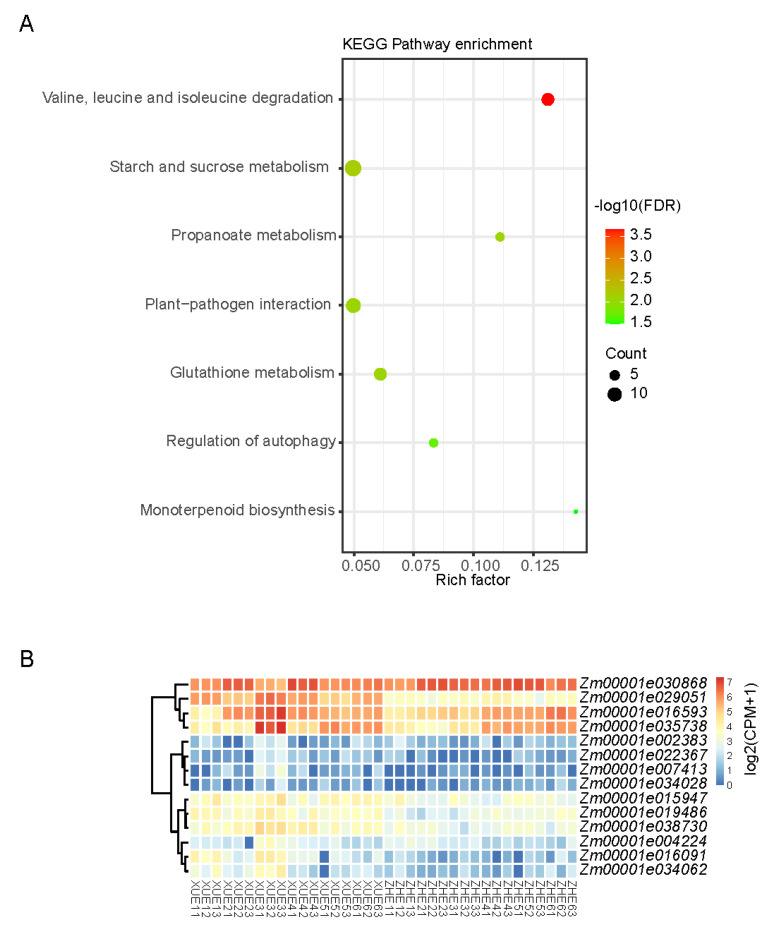
KEGG enrichment of MEyellow module genes (**A**) and heatmap of each sample expression level of starch and sucrose metabolism genes (**B**).

Genes in the MEyellow module were enriched in the KEGG pathway in response to starch and sucrose metabolism, which might influence sugar accumulation in kernels (Figure 7A); however, none of the related pathways were enriched in the other three modules. The expression levels of fourteen genes in the MEyellow module, which were enriched in the starch and sucrose metabolism pathway, were analyzed. The heatmap shows that *Zm00001e035738* was significantly upregulated in Xuetian 7401 18 DAP (Figure 7B). The function of the gene was annotated as beta-glucosidase (EC: 3.2.1.21), which is involved in the degradation of polysaccharides and increases monosaccharide content in kernels.

## 4. Discussion

Sweet corn is a kind of special corn that is formed due to the accumulation of saccharides caused by gene mutations in the starch anabolism of normal corn [24]. The sugar content of the kernel is an important phenotype of sweet corn. However, the mechanism of sugar accumulation in the kernels of sweet corn is still unclear. Here, dynamic changes in WSP contents in developing kernels, as well as some genes related to sugar accumulation in kernels, were revealed using genome-wide transcriptome analysis. 

The dynamic changes in the WSP content in the developing kernels showed that Xuetian 7401 transferred more sucrose to kernels, which is an important factor in maintaining the high level of sugar content. As sucrose is the main product of photosystem transfer in cereals [25], the rate of sucrose accumulation in kernels in the milking stage reflects the photosynthetic efficiency to some degree. On the other hand, the D-fructose content in Xuetian 7401 is much higher than in Zhetian 11, which contributes to the sweetness of Xuetian 7401, as D-fructose is 1.5 times sweeter than sucrose. According to the dynamic changes in the WSP content, the photosystem of Xuetian 7401 might be more efficient than that of Zhetian 11; meanwhile, the metabolism rates of D-fructose and D-glucose in Xuetian 7401 might be slower than those in Zhetian 11. With the speediness of anabolism and the slowness of the metabolism of sugar, this might be the reason why Xuetian 7401 is much sweeter than Zhetian11. Thus, the transcriptomes of the photosynthetic system and fructose metabolism were analyzed emphatically.

In this study, the important DEG-encoding photosynthetic system II subunit S (PsbS) was found in leaves, and this might influence sugar accumulation in sweet corn, as it is known that photosynthesis produces sugar, carbohydrates, lipids and proteins using light, water and carbon dioxide. Green plants contain two sets of photosynthetic systems, photosystem I (PS I) and photosystem II (PS II), each of which consists of a reaction center, a core antenna complex and a peripheral antenna complex. The function of PS II is light harvesting and splitting water into molecular oxygen, electrons and protons [26]. Structural observation showed that each PS II complex was composed of over 20 subunits and 77 cofactors [27]. PS I electrons were transferred through plastoquinones by PS II, which produced ATP and NADPH simultaneously [28]. PsbS was first identified in spinach [29], and the amino acid sequence showed that this protein belonged to the light-harvesting complex (LHC) protein superfamily [30,31]. Different from other LHC proteins, with three transmembrane helixes (TMN), four TMNs have been observed in PsbS [32]. PsbS also differed from ordinary LHC proteins in function, in that they are necessary for nonphotochemical quenching but not for efficient light harvesting and photosynthesis [33]. It was reported that PsbS influences the yield of the plant. The *PsbS* knockout mutant of *Arabidopsis thaliana* produces 30–50% fewer seeds than the wild type [34]. On the contrary, overexpressed *PsbS* in tobacco leads to a 15% increase in dry mass [35]. Here, we found two transcripts of *PsbS* in the leaves of Xuetian 7401 and Zhetian 11, and the expression levels were different in the two cultivars. *ZEAMMB73*_*Zmo0001d042697_1* was barely found in Zhetian 11, and the transcript lacked two regions (20 bp for each region) compared with *ZEAMMB73_Zmo0001d042697* that were found in both cultivars. The divergences in the sequences and expression levels of PsbS in the two cultivars might influence sugar accumulation in kernels.

Hexokinases (HXKs, EC: 2.7.1.1) are necessary for plants, as the hexokinase reaction represents the first step in the incorporation of hexoses into respiratory and biosynthetic pathways [36]. Except for the phosphorylation of hexose [37], HXKs also play important roles in signal transduction [38], growth and development regulation [39], pore control [40,41], mineral element absorption [42] and stress resistance [43]. In particular, HXKs play a role as key enzymes that limit the rate of the glycolytic process pathway [37]. *ZmHXK* was found to be involved in the processes of phytohormone and abiotic stress responses; sugar repression; light and circadian rhythm regulation; Ca^2+^ responses; seed development and germination; and CO_2_-responsive transcriptional activation [44]. However, it has not been reported that *ZmHXK* influences sugar accumulation in the kernels of maize. Starch and hexose contents were lower, but organic and amino acid contents were higher in the transgenic fruit of tomatoes with overexpressed HXK genes [45]. The activity of HXK in strawberries was found to be negatively correlated with the kernel sugar content of the fruit [46]. Similarly, the WSP content in fruits was significantly decreased compared with the wild type when *PbHXK1* was expressed in tomatoes [47]. However, there has been no similar report on maize. As the genes in cluster 7 were more downregulated in the kernels of Xuetian 7401 than in Zhetian 11 from 10 DAP to 30 DAP (Figure 4A), HXK encoded by *Zm00001e019856* was also downregulated in Xuetian 7401 and might contribute to sugar accumulation in kernels.

Some modules correlated with D-fructose and D-glucose were identified using WGCNA in this study, and the genes of the MEyellow module enriched the pathway of starch and sucrose metabolism. Furthermore, the expression level of *Zm00001e035738* in the kernels of Xuetian 7401 was significantly upregulated at 18 DAP (Figure 7B). The gene was annotated as beta-glucosidase, hydrolyze β-D-glucoside, cellodextrin and cellobiose by D-glucose in the starch and sucrose metabolism pathway. Beta-glucosidases are glycosyl hydrolases that hydrolyze the β-O-glycosidic bond of the anomeric carbon of a glucose moiety at the nonreducing end of a carbohydrate or glycoside molecule [48]. It was found that the D-fructose and D-glucose contents in the kernels of Xuetian 7401 reached a peak at 18 DAP; meanwhile, the expression level of *Zm00001e035738* was also the highest on the same dates from 10 DAP to 30 DAP. Considering the function of the gene and high connectivity between the MEyellow module and the kernel sugar content (Figure 6B), the high regulation of *Zm00001e035738*-encoding beta-glucosidase at 18 DAP might be helpful in producing more D-glucose in the kernels of Xuetian 7401.

In summary, the genes that encode PsbS and HXK were found in this study using genome-wide transcriptome analysis, which involved sugar anabolism and metabolism in maize. The variety of PsbS transcripts and the upregulation of beta-glucosidase in Xuetian 7401 might be helpful in producing more D-fructose and D-glucose in kernels, and the downregulation of HXK might contribute to slowing down the consumption of sugar. The results supplied a new insight into breeding new corn cultivars with high kernel sugar content and also laid a foundation for exploring the regulation mechanism of corn kernel sugar content. The functions and regulation mechanisms of the genes in sweet corn remain to be studied. 

## Figures and Tables

**Table 1:** Sequences of primers used in qRT-PCR.

Primer	Sequences (5′-3′)
*CyanateF*	GCTGGTGAGGAGGAGAAACA
*CyanateR*	CAGCAATCATGCCAGGTAGA
*ONT.1.1-F*	GAGTCCTCTGGAAGCTCTGTG
*ONT.1.1-R*	GGAGCAGCATCAAACAACCTG
*ONT.10003.1-F*	ATCCAATGCTGGGGGTGATG
*ONT.10003.1-R*	GATGGAGAGGGTATGCGAGC
*ONT.10030.1-F*	ACTTGGTTTGCCACCTTTGC
*ONT.10030.1-R*	GAATTGCTGCGGATTGGACC
*ONT.10032.2-F*	TTCCAGGAGCACTTCGATGC
*ONT.10032.2-R*	AGTCTAGTCGAGGCCGAAGT
*ONT.10200.1-F*	AGCGTATCCGTCTGAGCAAC
*ONT.10200.1-R*	GCCTCCAATGCACGTTCAAG
*ONT.11355.1-F*	GTTGTCGTTCTGTTCACCGC
*ONT.11355.1-R*	TTCAGGTTCGTTCACCAGCA
*ONT.11394.1-F*	CTCGGATCGTCGTCTCATGG
*ONT.11394.1-R*	AAGTCCACCACCTTGGGTTC

**Table 2:** Statistical table of the DEG numbers of developing kernels.

DEG Set	DEG Number	Upregulated	Downregulated
Xuetian 7401 (10 DAP) vs. Xuetian 7401 (14 DAP)	2636	1352	1284
Xuetian 7401 (10 DAP) vs. Xuetian 7401 (18 DAP)	3693	2209	1484
Xuetian 7401 (10 DAP) vs. Xuetian 7401 (22 DAP)	3057	1545	1512
Xuetian 7401 (10 DAP) vs. Xuetian 7401 (26 DAP)	4169	2069	2100
Xuetian 7401 (10 DAP) vs. Xuetian 7401 (30 DAP)	4596	2374	2222
Zhetian 11 (10 DAP) vs. Zhetian 11 (14 DAP)	1186	576	610
Zhetian 11 (10 DAP) vs. Zhetian 11 (18 DAP)	2207	1409	798
Zhetian 11 (10 DAP) vs. Zhetian 11 (22 DAP)	1760	986	774
Zhetian 11 (10 DAP) vs. Zhetian 11 (26 DAP)	2265	1177	1088
Zhetian 11 (10 DAP) vs. Zhetian 11 (30 DAP)	2576	1530	1046
Zhetian 11 (10 DAP) vs. Xuetian 7401 (10 DAP)	3552	1630	1922
Zhetian 11 (14 DAP) vs. Xuetian 7401 (14 DAP)	2519	1282	1237
Zhetian 11 (18 DAP) vs. Xuetian 7401 (18 DAP)	4036	2189	1847
Zhetian 11 (22 DAP) vs. Xuetian 7401 (22 DAP)	2147	999	1148
Zhetian 11 (26 DAP) vs. Xuetian 7401 (26 DAP)	2862	1457	1405
Zhetian 11 (30 DAP) vs. Xuetian 7401 (30 DAP)	2487	1144	1343
Total	45,748	23,928	21,820

## Data Availability

Data are contained within the article or Supplementary Materials.

## Supplementary Materials

The following supporting information can be downloaded at https://www.mdpi.com/article/10.3390/metabo12121254/s1: Figure S1: Principal components analysis (PCA) of ear and leaf samples; Figure S2: GO enrichment of leaf DEGs; Figure S3: KEGG enrichment of leaf DEGs; Figure S4: KEGG enrichment of MEbrown, MEmidnight blue and MEred modules; Table S1: Clean data statistics table of 40 samples.
