# Supplementary figures and images for: Genome-Wide Transcriptome Analysis Revealing the Genes Related to Sugar Metabolism in Kernels of Sweet Corn
Source: Metabolites. 2022 Dec 12;12(12):1254. doi: 10.3390/metabo12121254 (PMC9785893; doi:10.3390/metabo12121254)

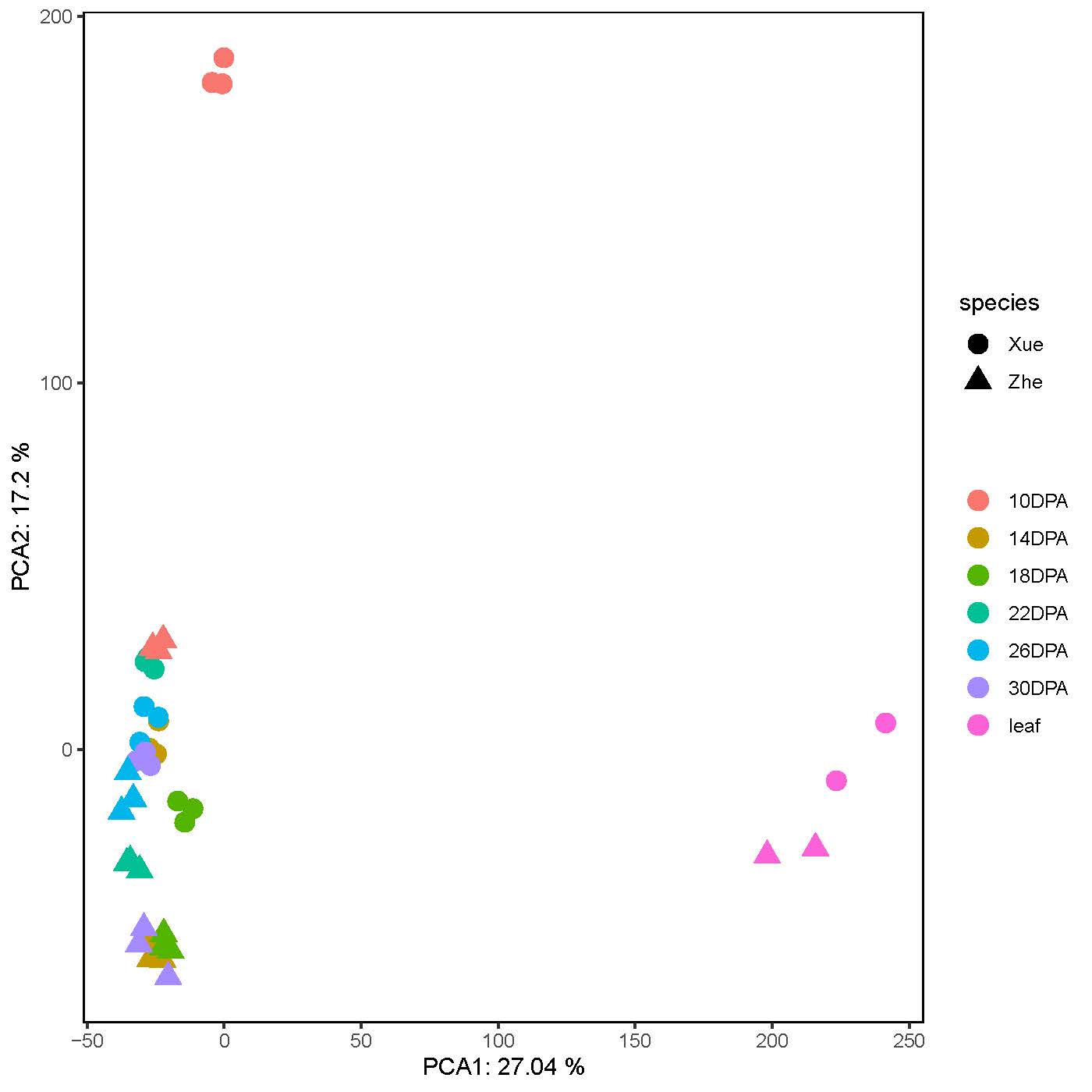

Supplement: Supplementary file 1 [file metabolites-12-01254-s001.zip › Figure S1.jpg]

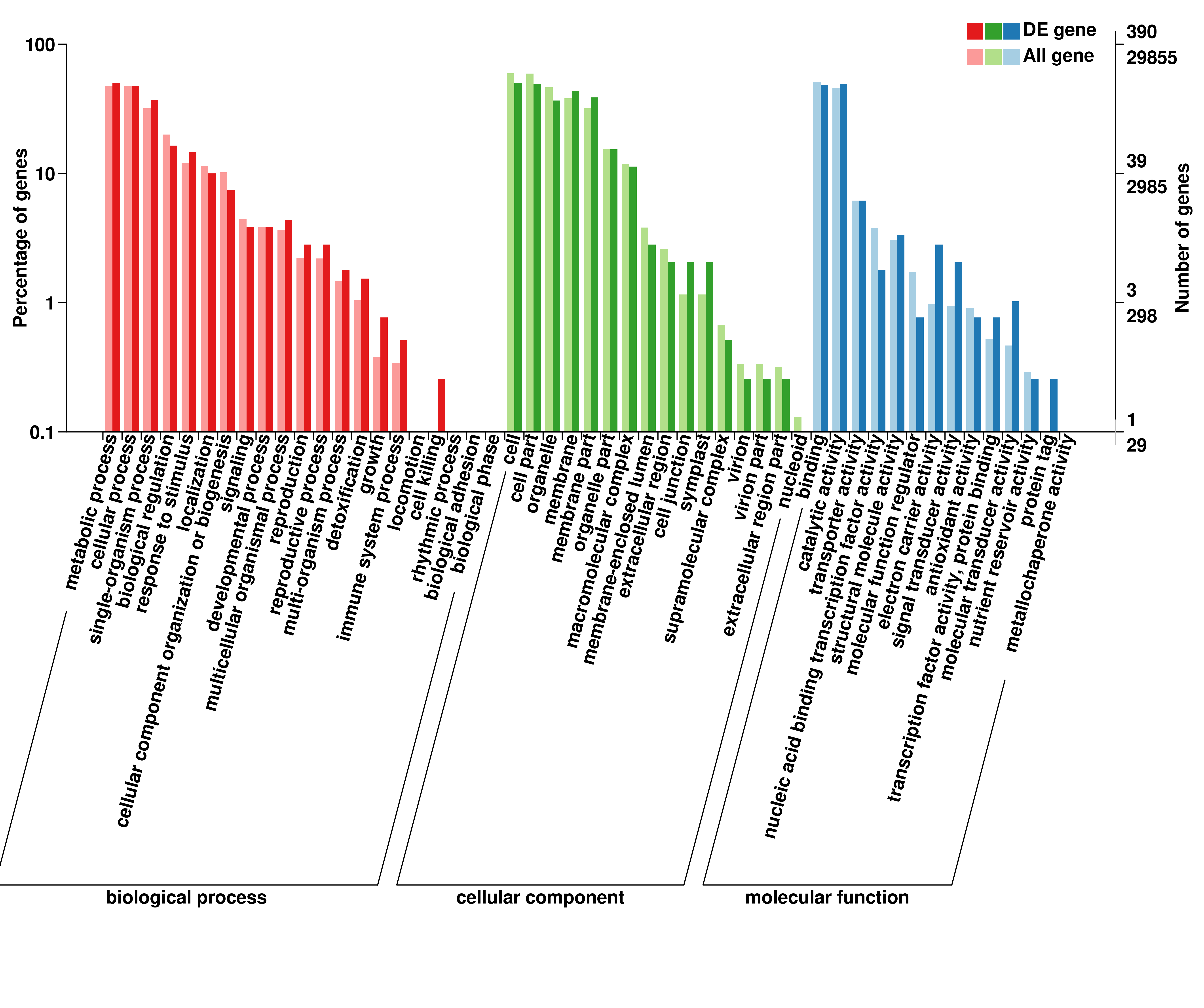

Supplement: Supplementary file 1 [file metabolites-12-01254-s001.zip › Figure S2.png]

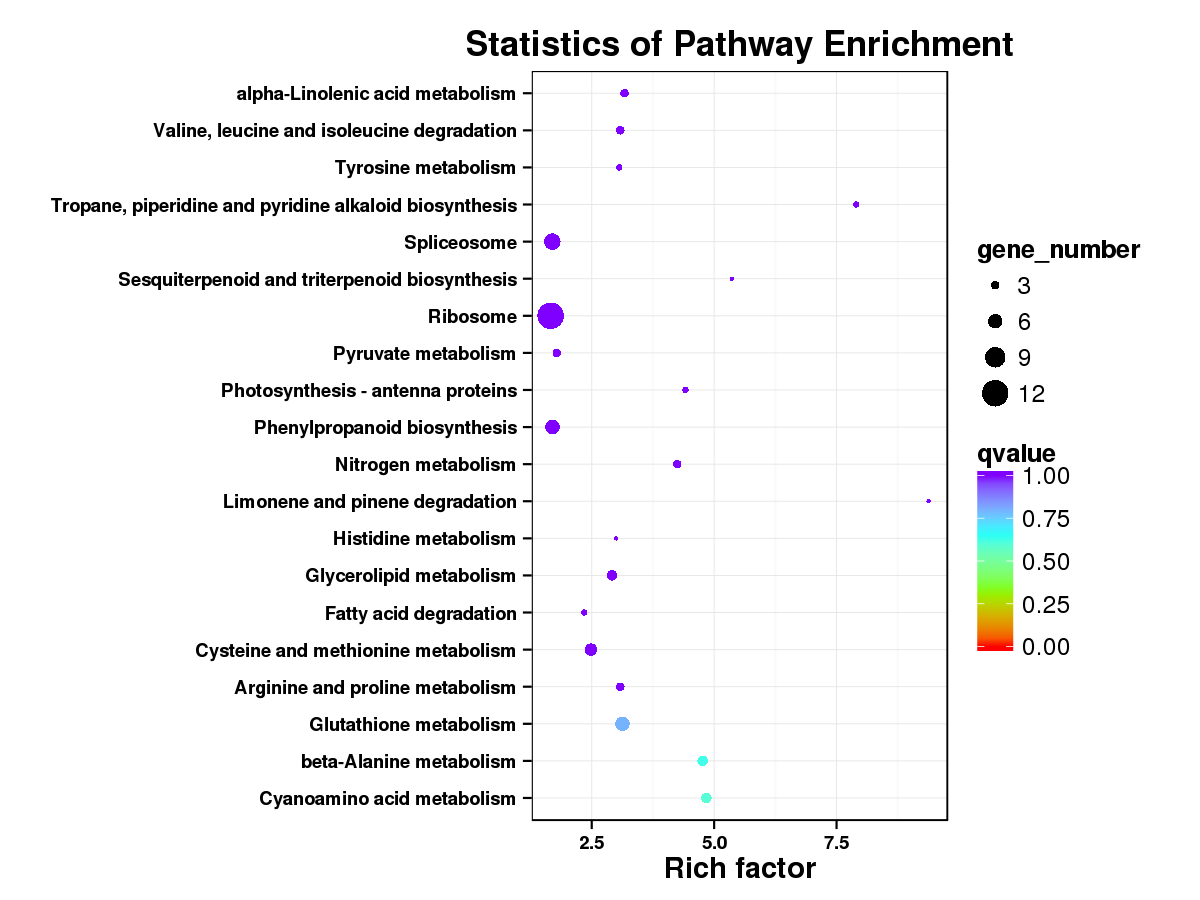

Supplement: Supplementary file 1 [file metabolites-12-01254-s001.zip › Figure S3.png]

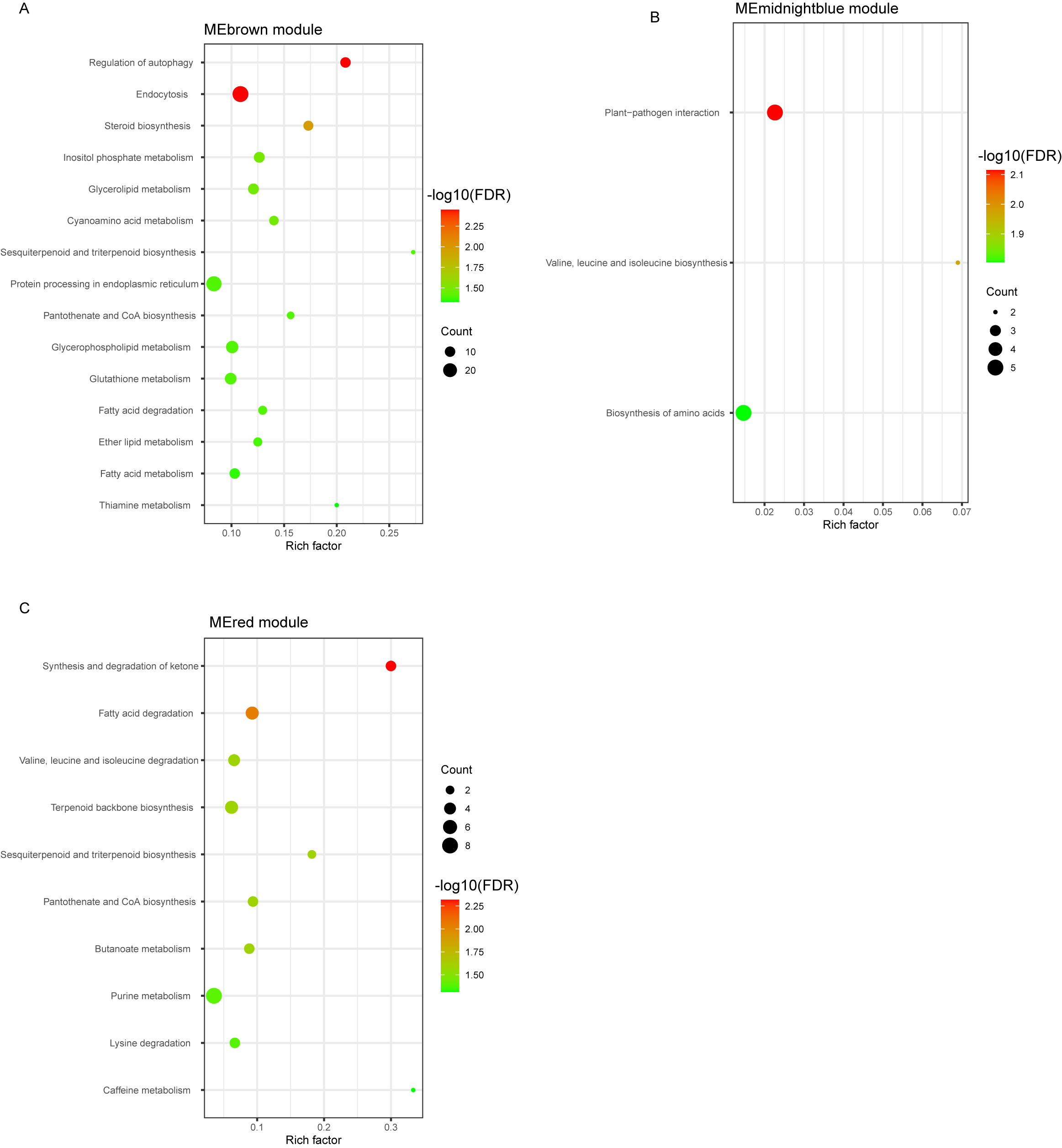

Supplement: Supplementary file 1 [file metabolites-12-01254-s001.zip › Figure S4.tif]
